# *In situ* rapid synthesis of hydrogels based on a redox initiator and persistent free radicals

**DOI:** 10.1039/d3na00038a

**Published:** 2023-03-07

**Authors:** Wei Yuan, Fangfang Wang, Xinyu Qu, Siying Wang, Bing Lei, Jinjun Shao, Qian Wang, Jianjian Lin, Wenjun Wang, Xiaochen Dong

**Affiliations:** a Key Laboratory of Flexible Electronics (KLOFE) & Institute of Advanced Materials (IAM), School of Physical and Mathematical Sciences, Nanjing Tech University (NanjingTech) Nanjing 211816 China chelseawq@njtech.edu.cn iamxcdong@njtech.edu.cn; b School of Physical Science and Information Technology, Liaocheng University Liaocheng 252059 China; c Key Laboratory of Optic-electric Sensing and Analytical Chemistry for Life Science, MOE, Shandong Key Laboratory of Biochemical Analysis, College of Chemistry and Molecular Engineering, Qingdao University of Science and Technology Qingdao 266042 China Jianjian_Lin@qust.edu.cn; d School of Chemistry & Materials Science, Jiangsu Normal University Xuzhou 221116 China

## Abstract

The development of fast and economical hydrogel manufacturing methods is crucial for expanding the application of hydrogels. However, the commonly used rapid initiation system is not conducive to the performance of hydrogels. Therefore, the research focuses on how to improve the preparation speed of hydrogels and avoid affecting the properties of hydrogels. Herein, a redox initiation system with nanoparticle-stabilized persistent free radicals was introduced to rapidly synthesize high-performance hydrogels at room temperature. A redox initiator composed of vitamin C and ammonium persulfate rapidly provides hydroxyl radicals at room temperature. Simultaneously, three-dimensional nanoparticles can stabilize free radicals and prolong their lifetime, thereby increasing the free radical concentration and accelerating the polymerization rate. And casein enabled the hydrogel to achieve impressive mechanical properties, adhesion, and electrical conductivity. This method greatly facilitates the rapid and economical synthesis of high-performance hydrogels and presents broad application prospects in the field of flexible electronics.

## Abstract 2

With a redox initiation system and stable persistent free radicals, hydrogels could be rapidly synthesized *in situ*. This approach greatly facilitates the rapid and economical synthesis of high-performance hydrogels.

## Graphical abstract

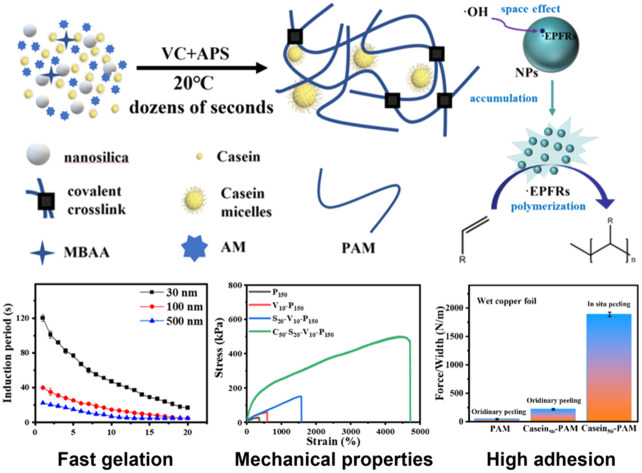

## 1. Introduction

Hydrogels have become very popular due to their unique properties, such as high water content, softness, flexibility, and biocompatibility. Natural and synthetic hydrophilic polymers can be physically or chemically cross-linked to produce hydrogels.^1–3^ Hydrogels are promising candidate materials for wearable flexible electronic devices due to their tissue-like high water-containing chemical structure, flexibility, and conductivity.^4–8^ Among them, tough hydrogels are widely used in various fields due to their excellent mechanical properties. Numerous studies have shown that free radical polymerization of vinyl monomers is a simple and versatile method for building tough covalent hydrogel networks.^9^ However, the initiation of free radicals usually requires external stimuli, such as thermal initiation or photoinitiation.^10–12^ Moreover, the method of polymerizing hydrogels by external stimulation requires specific production occasions, which not only consumes a lot of time and energy, but also limits the specific applications of hydrogels in bioelectronics, coatings, and other fields.

To realize the wide application of hydrogels, the great challenges in material design and structures are as follows: (i) *in situ* self-aggregation without external stimuli, (ii) rapid and adjustable polymerization and crosslinking reactions, (iii) long-lasting adhesion, (iv) excellent mechanical stability and toughness, and (v) using raw materials that are easy to synthesize and preferably available on the market. To solve the problem of initiation conditions in the preparation of hydrogels, rapid *in situ* synthesis strategies based on redox initiator systems have become an important research field in recent years. Redox systems typically use reducing agents and persulfates to rapidly generate free radicals at room temperature to initiate polymerization. In traditional redox initiation systems, reducing agents usually include inorganic low-valent transition metal ions and organic tetramethylethylenediamine.^13^ However, the reaction of low-valent metal ions and persulfates is too rapid to obtain a uniform hydrogel, and tetramethylethylenediamine is irritating to the skin and eyes, which is not conducive to the application of hydrogels in flexible devices and medical fields. In recent years, the metal phenolic network has gradually become a research hotspot.^14–17^ The system is composed of dopamine, tannic acid, lignin, and some other quinol-containing molecules and high-valent transition metal ions, which form an oxidation-reduction initiating system and reduce persulfates at room temperature to generate free radicals. However, this system still has shortcomings that need to be overcome. Since radical polymerization is an oxidative process, excess low-valent metal ions or phenolic species can inhibit the polymerization of monomers, resulting in failure of the gelation process or destruction of the mechanical properties of the obtained hydrogels. This requires an excess of high-valent metal ions to maintain the oxidation process in the system, which in turn leads to over-crosslinking of the polymer matrix or over-oxidation of phenols, resulting in a sharp decrease in the adhesive strength of the hydrogel. At present, the realization of fast-gelling viscous hydrogels with tunable gel time still faces great challenges.

Persistent free radicals discovered in environmental science provide new ideas. The life span of free radicals is usually only a few picoseconds.^18–20^ In 1977, Heimer discovered that free radicals can persist in the reaction of ethylene tetracyanide with subsulfonamide for the first time, and this free radical with certain stability and persistence was called a persistent free radical.^21^ Subsequently, more studies have shown that persistent free radicals can exist in many media, such as particulate matter, carbon fiber, and petroleum coke, with lives ranging from minutes to days.^22–25^ The stability of persistent free radicals mainly comes from the repulsive electron induction effect, conjugate effect, and spatial effect, which will increase the stability of free radicals. It is worth noting that a large number of literature studies have also shown that the combination of free radicals and nanoparticles can further stabilize free radicals to persist in the environment.^26–28^ The combination of a redox initiation system with stable persistent free radicals of nanoparticles may pave a new way to solve the problem of rapid preparation of hydrogels without sacrificing their mechanical properties.

Casein is the main protein component of milk. It is composed of a family of related phosphoproteins of αs1-, αs2-, β- and κ-casein, and their relative amounts are approximately 4 : 1 : 3.5 : 1.5.^29^ Casein has many beneficial properties suitable for biological materials, such as good biodegradability and biocompatibility.^30,31^ κ-Casein is a hydrophobic compound presented on the surface of casein micelles, which enables casein to form a micellar structure in a hydrated state.^4,32^ The product of the redox initiator, SO_4_^2−^, as a strongly hydrated ion, can reduce the solubility of casein due to the Hofmeister effect: the interaction of macromolecules and ions removes the water of hydration from the protein and folds the protein, prompting casein to form a three-dimensionally (3D) cured structure through hydrophobic association.^33^ In particular, casein micelles are composed of many sub-micelles that can be rearranged by applying shear force. Through this rearrangement of submicelles, casein micelles may undergo plastic deformation and thus show high potential for energy dissipation.^34,35^ In addition, casein micelles can act as physical cross-linking points in hydrogels to enhance their mechanical properties. With possible abundant physical interactions, including hydrogen bonding, hydrophobic interactions, metal complexation, and electrostatic interactions, casein will endow hydrogels with promising adhesion properties.^36,37^

**Scheme 1:**
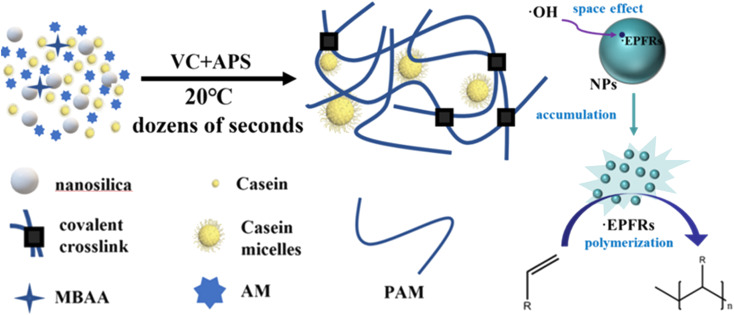
Mechanism of fast polymerization based on a redox initiator system and persistent free radicals.

Herein, we developed a strategy to self-initiate and produce tough hydrogels in one step in tens of seconds. This advanced fabrication strategy included a redox initiator system, nanoparticle-based persistent free radicals, and casein-enhanced mechanical and adhesive properties to establish a tough and sticky hydrogel network. The novel hydrogel synthesis system consisted of VC, ammonium persulfate (APS), nano-silicon dioxide (NSD), casein, *N*,*N*′-methylene diacrylamide (MBAA), and acrylamide (AM). Among these, VC-APS acted as a redox initiator to generate free radicals and NSD acted as a free radical stabilizer to accelerate gelation (Scheme 1). In this way, a high-performance casein-polyacrylamide (casein-PAM) hydrogel can be fabricated within 1 min at a mild temperature of 20 °C. In this system, NSD promoted the stability of free radicals, prolonged the existence time of free radicals, and facilitated the accumulation of free radical concentration in the hydrogel precursor. By adjusting the amounts of VC and NSD, the polymerization speed of the hydrogel can be adjusted to achieve special applications. In addition, based on the micellar structure of casein and various physical interactions, casein-PAM hydrogel also exhibited high stretchability, high strength, and high reversible adhesion. This preparation strategy is beneficial for the production of hydrogels in various flexible electronic fields and is expected to boost the mass production of wearable electronic devices.

## 2. Experimental section/methods

### 2.1 Materials

Casein (>90% dry basis), VC (99% RG), tetraethyl orthosilicate (TEOS) (≥99% RG), DMPO (98%+ RG), ammonium hydroxide solution (29% RG), AM (≥99.5% RG), APS (98% RG), and MBAA (99% RG) were purchased from Sigma-Aldrich (St Louis, MO, USA). All chemicals were used as received unless otherwise stated.

### 2.2 Preparation and characterization of silica nanoparticles

A mixture of 3 ml ammonia solution and 75 ml ethanol was dissolved in 10 ml water. After stirring at 30 °C for 5 min, 4 ml TEOS was added rapidly and stirred at room temperature for 1 h. After the reaction, the solution was centrifuged and washed three times to remove the unreacted TEOS and ammonia, and then washed three times with ultrapure water to remove ethanol. NSDs with different particle sizes were obtained by adjusting the amount of ammonia solution. The morphology and particle size of NSDs were characterized by using SEM and a particle size analyzer.

### 2.3 Electron spin resonance spectroscopy (ESR) analysis

ESR analysis was performed on an ESR spectrometer (JES-FA200 ESR Spectrometer, Japan) at 9.873 GHz. To ascertain the free radicals in this system, 1 ml of VC (0.02 g ml^−1^) and 1 ml of APS (0.02 g ml^−1^) solutions were mixed. Subsequently, 1 ml of DMPO (0.02 g ml^−1^) was added to the mixture. The solution was rapidly transferred to a standard capillary and placed in an EPR spectrometer. To determine the type of radicals, the *g*-values were calculated according to the following equation:1
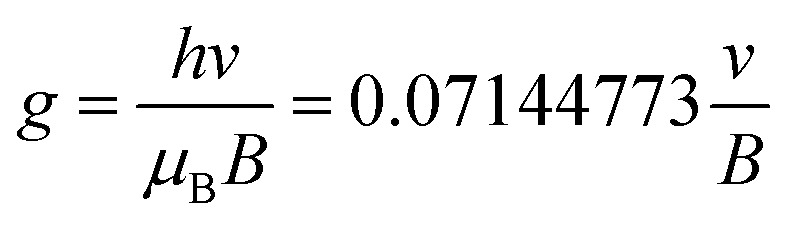
where the parameter *h* is the Planck constant, *v* is the frequency of the electromagnetic radiation, *B* is the magnetic field, and *μ*_B_ is the Bohr magneton.

### 2.4 Hydrogel preparation and gelation time characterization

First, AM and MBAA aqueous solutions with concentrations of 0.3 g ml^−1^ and 0.003 g ml^−1^ were prepared. According to the test requirements, appropriate APS and nano-silica dispersions were added to obtain uniform solution A. In addition, an appropriate amount of VC powder was added to deionized water and stirred to prepare solution B. Finally, solution A and solution B were mixed in a Petri dish (*Φ* = 55 mm) to form glue. The macroscopic fluid state in the reaction process was observed by the mechanical inversion method, and the temperature of the mixed solution was observed using an E50 infrared camera.

A rheological test was used to characterize the gelation time. All rheological tests were performed on a rotary rheometer. First, the hydrogel precursor solution (without VC) was injected into a plate with a gap of 1 cm (*φ* = 50 mm), and then the rotor was slowly decreased and evenly injected into the VC solution until the rotor was attached to the solution surface. The storage modulus (*G*′) and loss modulus (*G*′′) were recorded. When *G*′ > *G*′′, the fluid was solid, and when *G*′ < *G*′′, the fluid was liquid. When the two parameters were equal, it was regarded as a gel state. Therefore, *G*′ = *G*′′ was treated as the gel point, indicating hydrogel gelation.

### 2.5 Adhesion measurement

A 180° peeling strength test was performed to measure the adhesive strength of the hydrogel to substrates of different materials. The hydrogel was cut into pieces of 60 × 10 × 2 mm. The tests were applied in two ways. In one group, the hydrogel was first prepared, then adhered to the surface of the substrate, and pressed at 500 g for 30 min. The other group applied the hydrogel precursor directly to the surface and gelled at the surface for 5 min. The sample was pulled to failure using a digital stretcher under ambient conditions at a crosshead speed of 1 mm min^−1^. The peeling strength was calculated by dividing the measured maximum load by the width when stretched.

### 2.6 Mechanical and electrical measurement

The mechanical properties of the hydrogel were characterized by tensile tests. The tensile tests were performed on a mechanical test platform consisting of three parts, a force gauge (M4-2) for real-time measurement, a semiconductor parameter analysis device (Keithley 4200-SCS) that can apply a fixed voltage to a hydrogel sensor and record the current in real-time, and a stretching platform (ESM303, Mark-10) driven by a stepper motor to apply tension to the sensor. For the tensile tests, the tensile speed was set at 60 mm min^−1^.

The electromechanical properties of the hydrogel sensor were tested using a custom-designed intelligent data acquisition system including a computer-controlled stepper motor, a stretchable platform, and a semiconductor analyzer (Keithley 4200-SCS). To measure the signals associated with human activity, the hydrogel sensor was attached to the featured epidermis and the electrode was connected to a semiconductor analyzer for synchronous detection.

## 3. Results and discussion

### 3.1 Redox initiator

**Fig. 1:**
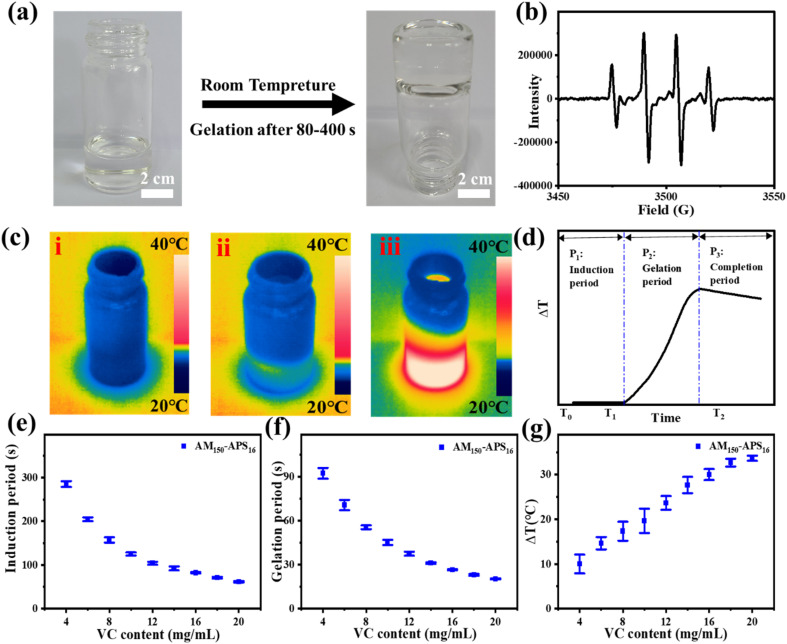
(a) Photograph of the rapid fabrication process of VC-PAM hydrogel at room temperature. (b) ESR spectra of pre-polymerization solution of the VC-APS redox initiator. (c) Thermographic images of the gel-forming process: (i) induction period, (ii) gelation period, and (iii) completion period. (d) Schematic diagram of the temperature–time curve of the gelation process. (e and f) Effects of the addition of different contents of VC on the induction time, gelation time, and temperature variation of silica-VC-PAM hydrogel (APS content: 16 mg ml^−1^).

VC and APS can form a redox pair, generating free radicals at room temperature to initiate the polymerization of the hydrogel precursor (AM-MBAA) within minutes (Fig. 1a). The advantage of this method is that it does not require any external stimulation and the oxidation–reduction process is relatively mild, non-toxic, and compatible. In the electron spin resonance (ESR) experiment, the free radicals generated by VC and APS in the aqueous solution are captured by 5,5-dimethyl-1-pyrroline *N*-oxide (DMPO). The ESR spectrum shows that the redox initiation system of VC-APS presents a quadruple signal with a relative intensity of 1 : 2 : 2 : 1, which is assigned to hydroxyl radicals (Fig. 1b).^38,39^ At room temperature, when VC and APS are added to the hydrogel precursor solution, the precursor quickly solidifies into a hydrogel (Fig. 1a).

The gelation process based on free radical polymerization mainly involves three basic steps: (i) initiation, (ii) growth, and (iii) termination. To explore the influence of VC content on the gel speed and temperature, the gel process is divided into three periods (Fig. 1c and d): the induction period (Fig. 1c(i)), the gelation period (Fig. 1c(ii)) and the completion period (Fig. 1c(iii)). The induction period is defined as the mixture dispersion time when the temperature remains constant. This period mainly involves the generation of free radicals and chain initiation reactions, which are crucial to controlling the overall polymerization rate. Gelation time is defined as the period during which the mixed solution temperature increases, and the chain growth reaction generally occurs during this period. The main characteristics of chain growth are exothermic and low activation energy, so the reaction speed is extremely fast. When the temperature reaches the peak, the chain radicals gradually lose their activity and bind to terminate the polymerization, forming a stable and uniform hydrogel, and this period is denoted as the completion period.

The reaction temperature is uniformly controlled at 20 ± 1 °C, and the effects of the amount of VC (AM 150 mg ml^−1^, APS 16 mg ml^−1^) on the induction period, gelation period, and temperature increase (Δ*T*) were evaluated. The polymerization reaction ended during the completion period, so it is not within the scope of the test. As shown in Fig. 1e and f, when VC content increases, the induction period and gel period shorten and the Δ*T* increases. When the VC content is lower than 4.0 mg ml^−1^, the polymerization reaction cannot take place. This is due to the extremely short lifetime of free radicals, and with inadequate VC content, the free radical generation rate is not sufficient to accumulate to reach a critical concentration to initiate polymerization. When the VC content reaches 4.0 mg ml^−1^, the polymerization occurs smoothly. The induction period is about 290 s, the gelation period is about 91 s, and the Δ*T* is about 10 °C. As the VC content increases to 20.0 mg ml^−1^, the induction period decreases to about 50 s, the gelation period is about 15 s and the Δ*T* increases to 35 °C. However, when the content of VC is further increased, limited by the content of APS, the induction period, the gelation period, and the Δ*T* will not change significantly. The VC-APS redox system can gently initiate polymerization, laying the basis for a controllable gel time with appropriate VC content.

### 3.2 Nanoparticles as free radical stabilizers

Nanoparticles could provide stability for free radicals through steric effects, prolong the lives of free radicals, and facilitate accelerating the accumulation of free radicals to quickly reach the critical concentration for initiating polymerization. Herein, different shapes of nanomaterials are added to the precursor to evaluate their effects on gelation time. Typical dimensions of nanoparticles, such as carbon nanotubes (CNTs, 1D) with similar chemical properties and different sizes, graphene (2D), and carbon black (CB, 3D) are chosen for comparative experiments.

**Fig. 2:**
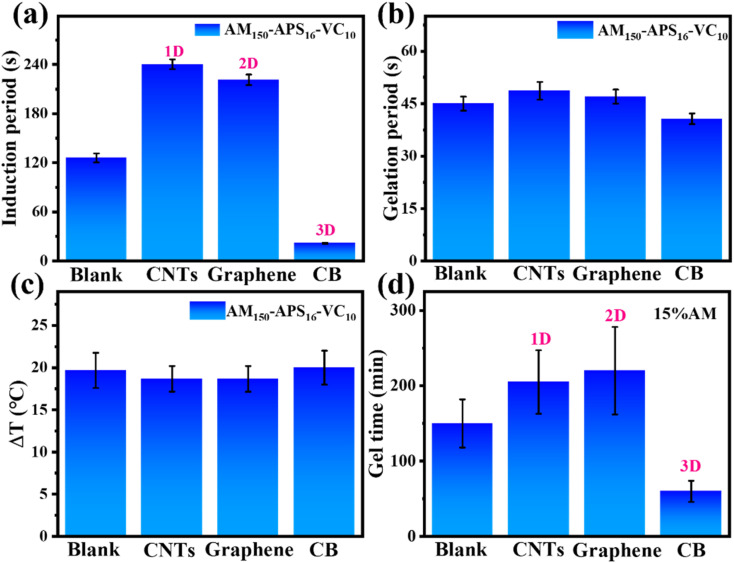
(a–c) Effects of the addition of nanomaterials (blank, CNTs, graphene, and CB) on the induction time, gelation time, and temperature variation of VC-PAM hydrogel. (d) Effects of adding nanomaterials (blank, CNTs, graphene, and CB) on gelation time under thermally initiated conditions of PAM hydrogel.

As shown in Fig. 2a–c, when different nanomaterials (2 wt%) are mixed into the redox-initiated VC-PAM hydrogel precursor, polymerization is initiated by VC at room temperature. The addition of CNTs (1D) or graphene (2D) greatly extends the induction period, while the addition of CB shortens the induction period from 126 s to 22 s, with a decrease rate of 82.5%. However, the time required for gelation and the temperature increase do not change much with the addition of different nanoparticles. The APS-initiated system in an oven (60 °C) also shows a similar trend as shown in Fig. 2d for thermally initiated PAM hydrogel. The addition of CNTs (1D) or graphene (2D) lengthens the curing time from 150 min to 210 and 225 min, respectively, while the addition of CB (3D) shortens the curing time from 150 min to nearly 60 min. The detrimental effect of 1D and 2D nanoparticles may be that their special shapes are not conducive to the spacing effect and steric hindrance effect. In addition, their large specific surface area provides abundant highly reactive sites, which makes it easy to quench free radicals and lead to low free radical concentrations.

**Fig. 3:**
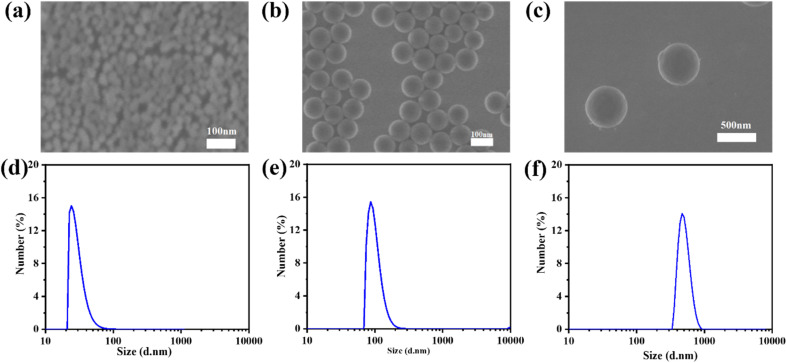
SEM and DLS characterization of (a and d) 30 nm, (b and e) 100 nm, and (c and f) 500 nm silica nanoparticles.

**Fig. 4:**
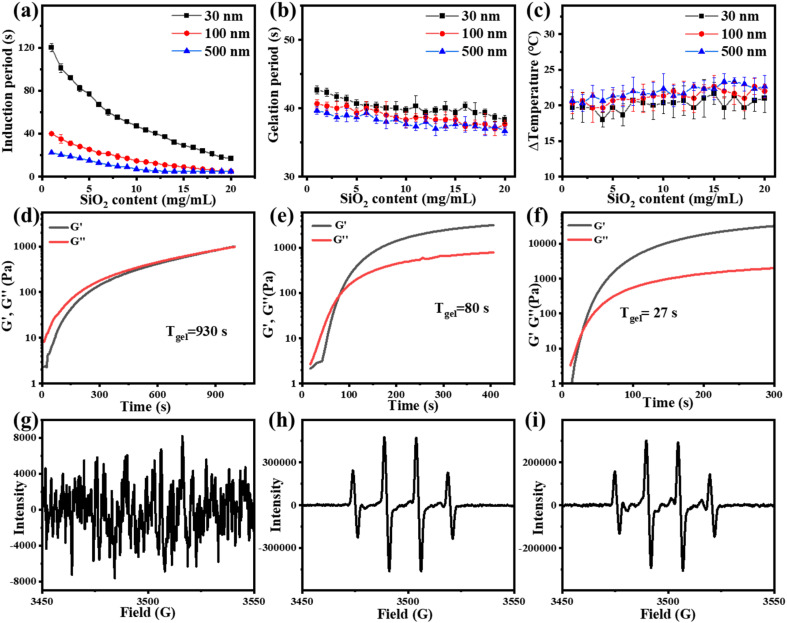
(a–c) Effects of the addition of different sizes and contents of nano-silica on the induction time, gelation time, and temperature variation of the silica-VC-PAM hydrogel. (d–f) Real-time *in situ* rheology of the VC-PAM hydrogel precursor at room temperature without stimuli (blank group, 30 nm and 500 nm). (g–i) ESR test of the VC-APS redox initiator system. (g) Blank group (before DMPO capture, reacting for 15 min). (h) Adding 100 nm nano-silica (before DMPO capture, reacting for 15 min). (i) Adding 100 nm nano-silica (before DMPO capture, reacting for 30 min).

To further study the influence of the size of three-dimensional nanoparticles on the gel process, NSDs with different sizes were synthesized. The scanning electron microscope (SEM) and dynamic light scattering (DLS) characterization studies reveal that the sizes of the NSDs are 30, 100, and 500 nm, respectively (Fig. 3). An infrared thermal imager was utilized to observe the temperature variation after mixing NSD with the VC-PAM hydrogel precursor (15% AM, 0.15% MMBA) and the results are displayed in Fig. 4a–c. When the amount of NSD increases, the induction period is significantly shortened (Fig. 4a), as well as a gentle decrease in the gelation period (Fig. 4b) and a negligible difference in Δ*T* (Fig. 4c) are observed. In addition, the induction period also shows a distinct time contraction with the increase in the size of NSD. The induction period of the 500 nm group needs about 25% of the induction period of the 30 nm group. It is identified that the specific surface area of the large-sized microspheres is decreased and the active sites are correspondingly reduced, thereby improving the stability of persistent free radicals. Moreover, large particles can provide more pronounced steric effects to stabilize the free radicals. The increase in persistent free radicals accelerates the accumulation of free radicals and therefore shortens the induction period. As shown in Fig. 4d–f, real-time *in situ* rheological studies are performed on hydrogel precursors of different compositions (blank, 30 nm, and 500 nm) to characterize the gelation process. In rheology, *G*′ is the storage modulus, which represents solid state properties, *G*′′ is the loss modulus that represents liquid properties, and the intersection of *G*′ and *G*′′ is generally regarded as the gel point. As shown in Fig. 4d–f, the *T*_gel_ of the blank sample is 930 s, for 2 wt% of the 30 nm nano-silica group, and *T*_gel_ is 80 s for the 2 wt% 500 nm nano-silica group, *T*_gel_ is further shortened to 27 s.

In the ESR experiment, the control group was set up and the blank group and the experimental group with 2 wt% 100 nm NSD were added respectively. After 15 or 30 min of reaction, DMPO was added to capture free radicals. As shown in Fig. 4g–i, in the group added with NSD, a clear hydroxyl radical signal could be detected after 15 min or even 30 min, while the blank group could not detect any significant signals. It can be seen that the nanoparticles could improve the stability of free radicals and significantly prolong the lifetime of free radicals.

### 3.3 Mechanical properties of hydrogels

**Fig. 5:**
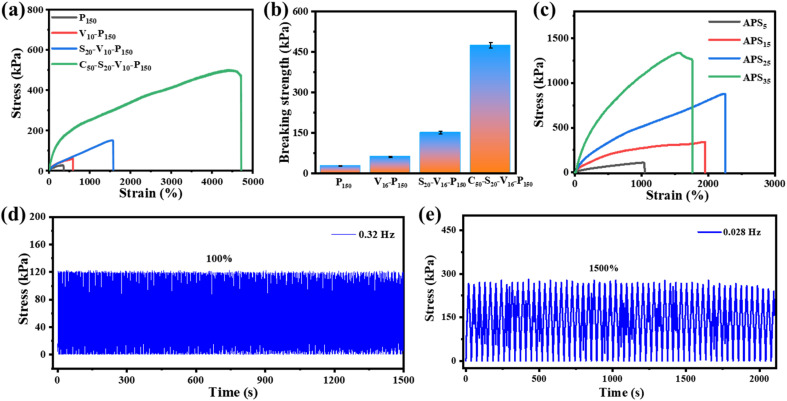
Mechanical performance of hydrogels. (a) Tensile stress–strain curves and (b) breaking strengths of hydrogels with different chemical components. (c) Tensile stress–strain curves of casein-PAM hydrogels after soaking (12 h) in different concentrations of APS solutions. The mechanical cycling performance of casein-PAM hydrogel under (d) tension at 100% strain and (e) 1500% strain.

Excellent mechanical properties are important prerequisites for hydrogel applications in various fields. Fig. 5a compares the mechanical properties of hydrogels with different initiator systems. It can be seen that the fracture tensile rate of PAM hydrogel induced by oven heating is only 360%, and the tensile strength is merely 26 kPa (Fig. 5b). PAM hydrogel is composed of rigid covalent bonds, and the crosslinking between polymer chains is irreversible chemical cross-linking. In addition, the long-term heating polymerization process can easily dehydrate the hydrogel and depress the chain mobility, so the obtained hydrogel is fragile. The fracture tensile rate and tensile strength of the VC-PAM hydrogel prepared using the oxidation–reduction initiator of VC-APS are greatly improved. Compared with high-temperature initiation, rapid polymerization at room temperature can retain water for the polymer matrix, maintain chain mobility in the hydrogel network, and make the hydrogel less prone to fracture. Nano-silica plays dual roles in nano-reinforcement and physical crosslinking and can be used as a filler to fill gaps and defects in hydrogel networks, effectively dissipate energy to prevent stress concentration and improve the mechanical flexibility of the hydrogel. In addition, based on the rapid gelation process of the redox system, nano-silica can be quickly anchored in the hydrogel network before agglomeration or settlement, which improves the uniformity of the polymer network. Moreover, the electrostatic interaction and hydrogen bond between nano-silica and polymer chains can also provide abundant reversible sacrificial bonds to enhance the mechanical properties of the hydrogel. When 2 wt% of 100 nm NSD is added, the breaking tensile rate of silica-VC-PAM hydrogel reaches 1170% and the breaking tensile strength increases to 151 kPa.

When 5 wt% casein is added into the precursor, the breaking tensile rate of the obtained casein-PAM hydrogel with a double network structure reaches 4710% and the breaking tensile strength exceeds 470 kPa (Fig. 5a). The introduction of casein with a folding structure provides a variety of synergistic physical interactions for polymer networks, which provides a large number of reversible sacrificial bonds in the polymer matrix and improves the energy dissipation capacity of the hydrogel. This can also form a dual network with PAM to improve the stretchability of the hydrogel. The non-covalent interaction between nano-silica and PAM long chains can further enhance the mechanical toughness of the hydrogel. Furthermore, SO_4_^2−^, the product of the APS and VC reaction, as a strong hydration ion, can remove the hydration water in the protein and prompt casein to spontaneously form micelles as the energy dissipation center. As evidenced in Fig. 5c, the mechanical properties of casein-PAM double-network hydrogel are significantly improved after being immersed in (NH_4_)_2_SO_4_ solutions for 12 h. However, with an increase in the (NH_4_)_2_SO_4_ solution concentration, the fracture tensile strength increases first and then decreases. With an increase in the salt solution concentration, the Hofmeister effect is enhanced, and more hydrophobic association chains are generated during the dehydration of the hydrogel, which increases the internal network density of the hydrogel and thereby enhances the tensile strength. When the concentration of (NH_4_)_2_SO_4_ solution is too high, the hydrogel will be plasticized due to severe dehydration, resulting in a decrease in fracture tensile rate.

The mechanical stability of the hydrogel in multiple stress loading–unloading cycles is of great significance for its functional application. As shown in Fig. 5d and e, uniaxial stress loading–unloading cycling tests at different tensions and stretching rates are carried out on the hydrogel. Casein-PAM hydrogel shows reliable mechanical stability in high frequency (100%, 0.32 Hz) and high tensile amplitude (1500%, 0.028 Hz) cycling tests. This is due to the permanent covalent crosslinking network provided by PAM and the reversible physical crosslinking network provided by nano-silica and casein.

### 3.4 Adhesive properties of hydrogels

**Fig. 6:**
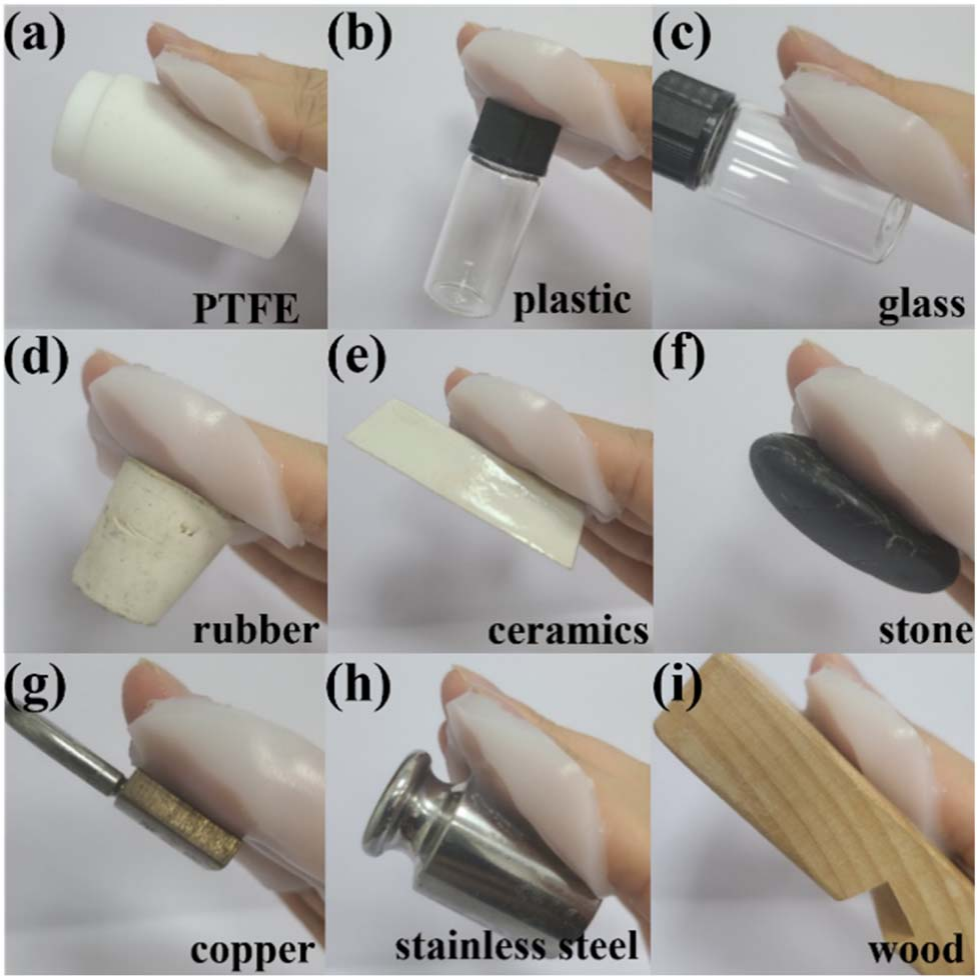
The adhesion properties of the casein-PAM hydrogel to different substrates.

The adhesion of the hydrogel is another prerequisite for its application in flexible sensors, dressings, coatings, and other fields. To vividly profile the adhesion performance of casein-PAM hydrogel, the digital photos of the hydrogel adhering to surfaces of polytetrafluoroethylene (PTFE), plastics, glass, rubber, ceramics, stone, copper, weight (stainless steel), and wood are shown in Fig. 6. Specifically, due to the low surface energy of PTFE, most of the hydrogels show poor adhesion to PTFE, while casein-PAM hydrogel can firmly adhere to the surface of PTFE.

**Fig. 7:**
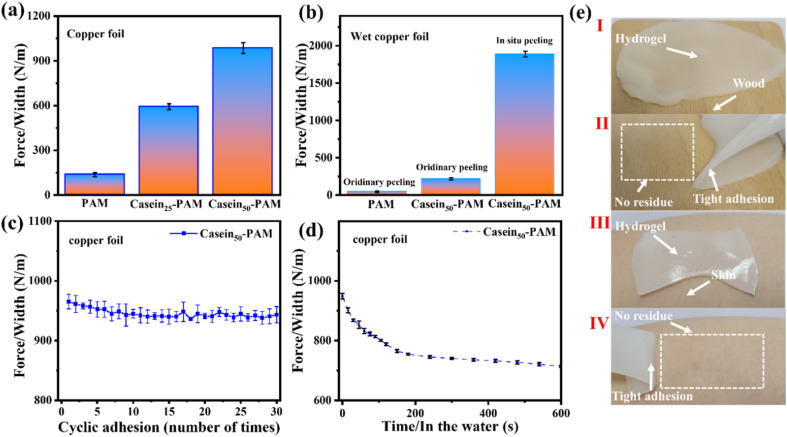
(a) Comparison of peel strengths of PAM, casein_25_-PAM, and casein_50_-PAM hydrogel. (b) Comparison of peel strength between ordinary adhesion and the *in situ* polymerized CP hydrogel on wet copper foil. (c) Peeling strength of hydrogel during cyclic adhesion. (d) Peeling strength of the hydrogel soaked in water after adhesion. (e) Digital photos of the hydrogel adhering to wood/skin and peeling from wood/skin.

The adhesion strength of casein-PAM hydrogel is evaluated by a 180° peeling test with copper foil as the adhesive substrate. As shown in Fig. 7a, with the introduction of different contents of casein, the peeling strength between the hydrogel and copper foil increases significantly. The peeling strength of pure PAM hydrogel is only 147 N m^−1^. When the casein content is 50 mg ml^−1^, the peeling strength can reach 985 N m^−1^. The enhancement in adhesion originates from the physical interactions provided by casein, including hydrogen bonding, hydrophobic interaction, metal complexation, and electrostatic interaction.^40^ It is worth noting that casein-PAM hydrogel exhibits higher adhesion when cured *in situ* on the substrate surface (Fig. 7b). Taking wet copper foil as an example, the adhesion strength of *in situ* curing increases from 217 N m^−1^ to 1886 N m^−1^, which is about 8.7 times higher than that of *ex situ* adhesion. This can be attributed to two aspects; on one hand, the *in situ* gelation has a higher conformal ability to the adhesive substrate and on the other hand, when *in situ* polymerization is performed on the surface of the substrate, the water on the substrate surfaces will combine with the groups in the hydrogel in the form of a hydrogen bond or van der Waals force, and become the bound water in the hydrogel, avoiding the destruction of adhesion by free water.

The durability of the hydrogel adhesion cycle and adhesion waterproofness are important parameters in its practical application. In the past, compounds containing catechol groups were often used to provide adhesion, but catechol is easily oxidized to quinone or semi-quinone to lose adhesion.^41^ As shown in Fig. 7c, after 30 adhesion–stripping cycles, the peeling strength of casein-PAM hydrogel remains about 95% of the initial peeling strength, showing excellent cycle adhesion stability and durability. When the casein-PAM hydrogel precursor is *in situ* gelled and adhered to the required substrate, the peeling strength remains above 70% of the initial strength even after 600 s of immersion in water (Fig. 7d). It can be seen from Fig. 7e that the hydrogel can adhere tightly to the surface of human skin or wood and be stripped without leaving a residue. These strong and reusable adhesive properties are beneficial for flexible wearable devices.

### 3.5 Electromechanical properties and sensing applications of the hydrogel strain sensor

**Fig. 8:**
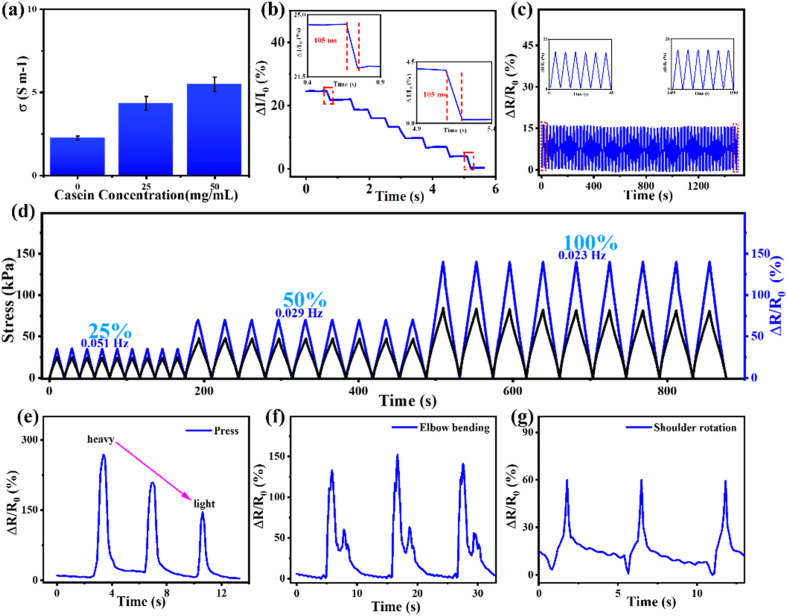
(a) Variation of the conductivity of hydrogels with the addition of different contents of casein. (b) Response time of the casein-PAM hydrogel sensor during stretching. (c) The durability test of the casein-PAM hydrogel sensor. (d) Cyclic stretching–releasing with different stretch frequencies, electromechanical hysteresis, and relative resistance variation of casein-PAM hydrogel under cyclic stretching-releasing at strains of 25%, 50%, and 100%. The sensing performance of the hydrogel sensor: (e) Pressing from heavy to light, (f) repeated bending of the elbow, and (g) repeated rotation of the shoulder.

The mechanical and electrical properties of hydrogels are the basis for their applications in flexible electronics, especially in flexible sensing. Due to the incorporation of casein, casein-PAM hydrogel contains abundant free ions to establish a smooth conductive network. As shown in Fig. 8a, the conductivity of hydrogels increases significantly with the introduction of casein. A typical casein-PAM hydrogel strain sensor displays excellent immediate response features to mechanical deformations. The hydrogel sensor shows a fast response (105 ms) to external stimuli (2% stretch, Fig. 8b), ensuring a real-time perception of target movement. As shown in Fig. 8d, the relative resistance changes are almost synchronized with the applied stress, indicating negligible electromechanical hysteresis in electromechanical perception. The relative resistance change of the casein-PAM hydrogel sensor reveals a distinct increase in amplitude in the stretching ranges of 25%, 50%, and 100% (Fig. 8d), indicating that the strain sensor has high reliability to monitor different degrees of tensile strain. Fig. 8d also shows the response signals of the casein-PAM hydrogel sensor under different frequency strains. It is observed that at different frequencies of 0.051, 0.029, and 0.023 Hz, the relative resistance changes of the sensor are reproducible and durable, suggesting typical frequency-dependent behavior. Fig. 8c demonstrates the relative resistance change of the casein-PAM hydrogel sensor during 1500 consecutive tensile stretching and releasing cycles at 0–10% strain. Typically, the enlarged curves inserted in Fig. 8c verify that the relative resistance changes are nearly constant throughout the test, indicating that the strain sensor exhibits excellent repeatability and durability.

Given that the casein-PAM hydrogel strain sensor possessed excellent mechanical properties, tough and reversible adhesion, excellent electromechanical properties, and stable electrical properties, the hydrogel sensor is suitable for directly attaching to human skin to monitor different amplitudes of body movement. When the hydrogel strain sensor is directly attached to the arm, it can accurately distinguish collision forces of different intensities (Fig. 8e). As shown in Fig. 8f and g, when the hydrogel sensor directly adheres to the elbow and shoulder joints, it can stably recognize the movements of different joints multiple times and show different peak shapes for different joint movements.

## 4. Conclusion

Casein-PAM hydrogel with self-polymerization, high tensile strength, and high adhesion was designed and synthesized by introducing a redox initiation system, the persistent free radical effect of nanoparticles, and a double network interpenetrating polymer strategy. The redox initiator system of VC-APS can spontaneously generate free radicals to initiate hydrogel polymerization at room temperature, and NSD acts as the free radical stabilizer to control the polymerization rate of the hydrogel. Chemically cross-linked PAM is the first crosslinking network to provide a rigid support for the hydrogel and the physically crosslinked casein constructs the second cross-linked network, which acts as a sacrificial bond to enhance the energy dissipation and improves the mechanical properties of the hydrogel. In addition, casein also provides abundant physical interactions to achieve tough adhesion for various substrates. The results showed that the casein-PAM hydrogel precursor could be rapidly gelled at room temperature, and the gelation time can be adjusted by adjusting the amount of the reducing agent and nano-silica. Hydrogels revealed excellent mechanical properties (fracture tensile rate 4710% and fracture tensile strength 470 kPa) and excellent adhesion, especially with the *in situ* gelation strategy on the wet material surface, which promised a wide application prospect. The fabricated sensor exhibited a stable electromechanical response and can be used as a flexible and wearable electronic device to monitor physiological signals of human motion.

## Conflicts of interest

There are no conflicts to declare.

## Supplementary Material
